# The Calcium Signalling Profile of the Inner Blood–Retinal Barrier in Diabetic Retinopathy

**DOI:** 10.3390/cells14120856

**Published:** 2025-06-06

**Authors:** Francesco Moccia, Silvia Dragoni

**Affiliations:** 1 Department of Medicine and Health Sciences “V. Tiberio”, University of Molise, 86100 Campobasso, Italy; francesco.moccia@unimol.it; 2 School of Applied Sciences, University of Brighton, Moulsecoomb, Brighton BN24GJ, UK

**Keywords:** diabetic retinopathy, blood–retinal barrier, endothelial dysfunction, calcium signalling

## Abstract

Diabetic retinopathy is a sight-threatening complication of diabetes mellitus, affecting millions of people worldwide. From a vascular perspective, diabetic retinopathy compromises the structure and function of the blood–retinal barrier, leading to aberrant angiogenesis and vascular leakage, with consequent loss of vision. This review will delve into the vascular abnormalities caused by diabetic retinopathy in the inner blood–retinal barrier, focusing primarily on retinal endothelial cells. It will then discuss how calcium signalling regulates inner blood–retina barrier function and dysfunction, how calcium channels contribute to the development of diabetic retinopathy, and how studying the components of the calcium toolkit may identify new therapeutic targets.

## 1. Introduction

Diabetes is a chronic metabolic disease characterised by elevated blood glucose concentrations, mainly related to impaired insulin metabolism [1]. While adipose tissue, skeletal muscles, and the liver are primarily affected by the disease due to insulin resistance, further vascular complications can also impact the heart, brain, and eyes [1,2].

Vision can indeed be significantly impaired when hyperglycaemia progresses to diabetic retinopathy (DR), a prevalent complication of diabetes mellitus [3,4]. DR dramatically affects the properties of retinal blood vessels, which supply the retina with oxygen and nutrients while protecting the neuronal environment from peripheral circulation [5]. Specifically, retinal capillaries form the blood–retinal barrier, where endothelial cells (ECs) are connected by tight junctions that restrict the passage of solutes and fluids between the bloodstream and the parenchyma [6,7]. In DR, the properties of ECs are compromised, thereby enhancing vascular permeability and promoting pathological angiogenesis [5,8].

The molecular mechanisms underlying DR have only been partially elucidated. Vascular endothelial growth factor (VEGF) is a key contributor to the vascular alterations observed in the disease. Under pathological conditions, elevated VEGF levels promote endothelial cell migration and proliferation, angiogenesis, extracellular matrix breakdown, and increased vascular permeability [9,10,11,12]. As a result, intravitreal anti-VEGF injections are the primary treatment for retinal disorders. However, up to 40% of patients show little to no response, and even among responders, resistance or adverse effects often arise, impacting treatment adherence [13,14]. Consequently, alternative therapeutic approaches are being actively explored.

As a universal intracellular messenger, calcium (Ca^2+^) regulates nearly every aspect of cellular life [15]. Both preclinical and clinical studies highlight that Ca^2+^ signalling becomes dysregulated in a wide range of pathological conditions, including diabetes and eye diseases [16,17,18,19]. EC functions are heavily dependent on Ca^2+^ channel activities and associated signalling pathways, which are altered in endothelial dysfunction, making them promising therapeutic targets [20,21,22]. Emerging evidence shows that the Ca^2+^ channel activity of several retinal cell types is dysregulated in hyperglycaemic conditions, contributing to the vascular alterations observed in DR [23]. This review will discuss the structure and function of the inner blood–retinal barrier (iBRB) in health and disease and describe the role of Ca^2+^ signalling in iBRB function and dysfunction, pointing to Ca^2+^ channels as emerging therapeutic targets for diabetic retinopathy.

## 2. Diabetic Retinopathy

**Figure 1:**
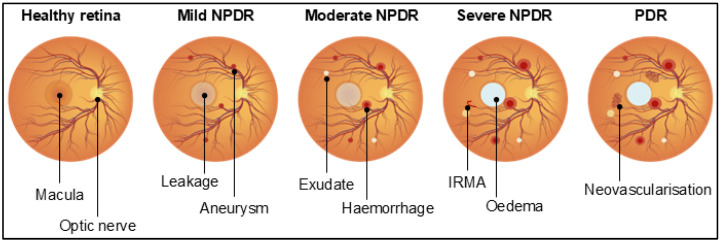
Vascular abnormalities in diabetic retinopathy. Retinae showing the progression of diabetic retinopathy and the vascular abnormalities associated with each phase of the disease. Non-proliferative diabetic retinopathy (NPDR), proliferative diabetic retinopathy (PDR), and intraretinal microvascular abnormality (IRMA). Created in BioRender. Dragoni, S. (2025) https://BioRender.com/kn96utz accessed on 14 April 2025.

DR is the most recurrent microvascular complication of diabetes mellitus [24], thereby representing a primary cause of vision impairment worldwide. The global prevalence of DR among people with diabetes is one-third, and it is estimated that the number of DR patients will rise from 103.12 million in 2020 to 160.50 million by 2045 [25]. From a vascular perspective, DR can be classified into non-proliferative diabetic retinopathy (NPDR) or proliferative diabetic retinopathy (PDR) [26]. NPDR is graded as mild when patients have at least one retinal microaneurysm, but no other findings; moderate when patients present haemorrhages or microaneurysms in one to three retinal quadrants and/or cotton wool spots, hard exudates, or venous beading; and severe when patients develop intraretinal haemorrhages (>20 in each quadrant), venous beading in two or more quadrants, or an intraretinal microvascular abnormality in one or more quadrants. All these stages are characterised by the absence of neovascularisation, which appears when NPDR progresses to PDR [4,26] (Figure 1). PDR is typified by neovascularisation in one or more regions of the eye, such as the iris, the angle of the anterior chamber, the optic disc or elsewhere in the retina, or by vitreous/pre-retinal haemorrhages [27]. Patients with PDR can also develop two types of fibrotic tissues, namely fibrovascular proliferative tissue and avascular proliferative tissue, which contribute to retinal detachment [28,29]. Different terms are then used to classify DR based on the location of microvascular abnormalities, and the presence and extent of vascular leakage. We can therefore observe diabetic maculopathy when the macula is affected and diabetic macular oedema (DMO), when vascular leakage accumulates and exacerbates into oedema. Although the diagnosis and classification of DR rely on vascular changes, as they are easy to visualise, it is important to highlight that the neuroretina functions start declining during the early stages of diabetes before the occurrence of vascular complications. Alterations include distorted colour vision [30], abnormal cone pathway response [31], loss of contrast sensitivity [32], and changes in the microglia [33,34].

## 3. The Inner Blood–Retina Barrier

**Figure 2:**
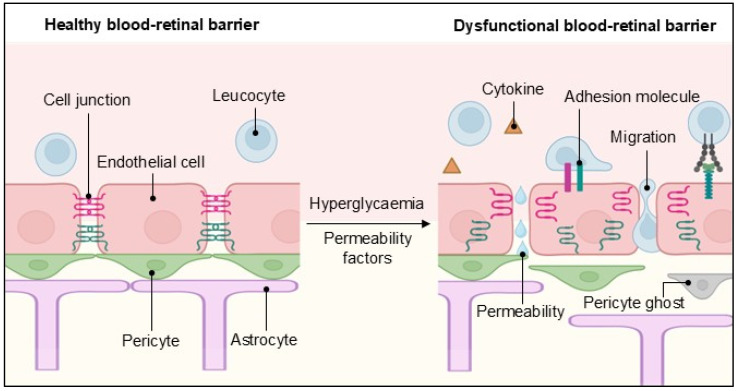
The inner blood–retinal barrier in health and disease. A schematic representation of the cellular components of the inner blood–retinal barrier and how they change during endothelial dysfunction, leading to vascular permeability, transendothelial migration and cytokine release. Created in BioRender. Dragoni, S. (2025) https://BioRender.com/8l6qtn9 accessed on 17 April 2025.

The iBRB is a set of capillaries that supply the retinal layers with oxygen and nutrients, and it is formed by ECs that tightly regulate the exchange of fluids, molecules, and cells between the bloodstream and the parenchyma [5,35,36,37]. ECs lie on a basement membrane and interact with astrocytes, pericytes, smooth muscle cells (SMCs), microglia, and neurons to form the neurovascular unit (NVU) [38] (Figure 2). Specialised properties of ECs minimise both paracellular and transcellular permeability. In the blood vessels of most tissues, ECs are held together by adherens junctions formed by vascular endothelial cadherin (VE-cad) and catenins [39,40,41]. However, in the brain and retina, tight junctions further reinforce these connections, establishing a high-resistance barrier that prevents paracellular transport while maintaining apicobasal polarity [6,42,43]. These tight junctions consist of claudins, MARVEL family transmembrane proteins (such as occludin, tricellulin, and MarvelD3), and junctional adhesion molecules (JAMs) [6,40]. Transcellular permeability at the iBRB is also tightly regulated. In retinal ECs, the transporter MFSD2A is overexpressed, limiting caveolin-mediated transcytosis, whereas plasmalemma vesicle-associated protein (PLVAP), which is important for fenestrae formation, is downregulated, limiting molecular transport across ECs [44,45,46]. Several efflux and solute transporters are highly expressed in retinal ECs. Among efflux transporters, the ATP-binding cassette superfamily G member 2 (ABCG2) and MDR1/P-glycoprotein (PGP), which are among the most abundantly expressed transporters in the iBRB, restrict the access of xenobiotics and exogenous molecules, including steroids. Essential nutrients are instead transported by solute transporters, such as GLUT1 and MCT1, down their concentration gradient, or through receptor-mediated vesicular transport, involving the transferrin receptor and low-density lipoprotein receptors [47,48].

Additionally, ECs regulate immune responses by expressing lymphocyte adhesion molecules, which mediate leukocyte binding and tissue infiltration. Under normal physiological conditions, ECs exhibit low levels of Intercellular Adhesion Molecule-1 (ICAM-1) and Vascular Cell Adhesion Molecule-1 (VCAM-1), and suppress cytokine-stimulated leukocyte adhesion to endothelium, for example, by secreting TGF-β, thereby minimising leukocyte migration into the retinal environment and preserving the iBRB immune privilege [49]. ECs lie on a basement membrane where pericytes are embedded. Pericytes are phagocytic cells that play a key role in vascular stability by influencing EC growth, vessel remodelling, and angiogenesis [50,51]. Pericytes can uptake fluids, macromolecules, or cell debris by phagocytosis, pinocytosis, and endocytosis [52]. Additionally, pericytes possess contractile properties that help regulate blood flow, a function essential for maintaining the iBRB [53,54,55]. In post-capillary venules, they contribute to immune regulation by expressing chemokines, cytokines, and adhesion molecules that facilitate leukocyte migration [56,57].

Communication among the NVU components is essential for maintaining its integrity. ECs interact with pericytes through gap junctions, membranous interdigitations called “peg-and-socket” connections, and paracrine signalling factors [58], and they share materials and exchange information by transferring microparticles and exosomes between each other. The communication between ECs and pericytes via several signalling pathways is required for angiogenesis. Those include platelet-derived growth factor B/platelet-derived growth factor receptor-β (PDGFB/PDGFR-β) signalling, which induces pericytes to proliferate and migrate toward ECs, thereby guiding pericyte recruitment, transforming growth factor-β/transforming growth factor-β receptor-2 (TGF-β/TGF-βR2) signalling, which is involved in angiogenesis and cell differentiation, as well as Notch pathway and Ang1/Tie2 signalling that regulate vascular maturation, stability, and remodelling [50,51,59]. During retinal development, ECs and pericytes interact with astrocytes to coordinate the expression of tight junction proteins [60].

Astrocytes, as key glial cells, further reinforce the iBRB by secreting inflammatory mediators and trophic factors while also regulating blood flow through neurovascular coupling. Their endfeet tightly envelop ECs, enhancing barrier integrity [61,62]. Astrocytes also contribute to neuronal health by regulating neurotransmitter release, such as glutamate and adenosine. They help maintain ionic and water homeostasis through mechanisms involving Ca^2+^ signalling, potassium and chloride channels, and aquaporin-4 [63,64]. During retinal vascular development, coordinated signalling between ECs, pericytes, and astrocytes promotes the expression of tight junction proteins necessary for iBRB integrity [60]. Additionally, the basement membrane is a fundamental element in NVU stability, and its thickening disrupts EC interactions with other NVU components, often serving as an early marker of retinal disease [65]. Finally, microglia—macrophage-like cells—are responsible for clearing cellular debris and releasing pro-inflammatory factors [66,67].

## 4. Endothelial Dysfunction in Diabetic Retinopathy

Disruptions to the NVU structure and function are key factors in the onset and progression of retinal disease. Indeed, during DR, the pericytes of retinal capillaries and the smooth muscle cells of arterioles undergo apoptosis, thereby destabilising the NVU and compromising iBRB integrity [68,69].

From a haemodynamic standpoint, changes in blood flow can be utilised as a diagnostic tool. Despite fluctuations in arterial or intraocular pressure, the retina maintains a constant blood flow through a mechanism known as retinal pressure autoregulation [70]. Impairments in pressure autoregulation may increase retinal capillary pressures, thereby causing shear-induced EC damage [71]. Consistently, abnormalities in retinal blood flow precede the early clinical stages of DR [72,73,74]. These correlate with changes in vessel calibre observed during the progression of DR, where arteriolar vasoconstriction is associated with the early stages of the disease, and is followed by a vasodilation typical of the later stages [75], therefore resulting in reduced and then enhanced blood flow [76], which may relate to the progression to diabetic macular oedema (DMO) and PDR [77,78]. To complete the picture, another critical contributor to the regulation of blood flow is the process of neurovascular coupling, which has been thoroughly discussed elsewhere [79]. In a streptozotocin-induced rat model of type 1 diabetes, abnormal neurovascular coupling contributed to the development of retinopathy through alterations in the nitric oxide (NO) signalling [80].

The breakdown of the iBRB occurs shortly after diabetes induction in animal models [42]. One of its first consequences is certainly vascular leakage, detectable by magnetic resonance imaging, even before clinically recognisable DR lesions appear [81]. The increase in paracellular permeability results from the disruption or decreased expression of cellular junction proteins, which allows fluids and immune cells to cross the barrier, thereby contributing to an inflammatory state (Figure 2).

iBRB breakdown is triggered by the activation of different molecular systems and pathways, including the plasma kallikrein–kinin system (KKS), VEGF, and pro-inflammatory cytokines and chemokines. Bradykinin is a potent vasodilator and a critical component of the plasma KKS [82]. The latter is a proteolytic pathway activated during vascular injury, contributing to inflammation, blood flow, and coagulation [83]. Bradykinin binds to the G protein-coupled receptors B1 and B2 in the plasma membrane of ECs. While B2 is constitutively expressed, B1 is upregulated in injury and inflammatory settings, such as DR [84]. When activated, both receptors lead to the production of NO and prostaglandins, with consequent vessel dilation [85,86]. Bradykinin can also activate Src kinases, leading to the phosphorylation of adherens junction protein VE-cadherin and reversible opening of EC junctions with consequent vascular leakage [87]. Indeed, intravitreal injection of bradykinin or B1 and B2 receptor agonists increased retinal vascular permeability in animal models [84]. Moreover, proteomic studies have identified components of the plasma KKS in the vitreous of patients with advanced DR [88,89].

VEGF, which contributes both to angiogenesis and vascular permeability, is also increased in the retina in DR [90]. Its expression is enhanced by hypoxia and causes a decrease in the expression of ZO1, phosphorylation, ubiquitination and subsequent inactivation of occludin via PKCβ, and phosphorylation of VE-cadherin [87], thereby increasing paracellular permeability.

Hyperglycaemic conditions trigger the synthesis of advanced glycation end products (AGEs) and reactive oxygen species (ROS), which activate the transcription factor NF-κB, with consequent expression of pro-inflammatory molecules [91]. Among those, TNF-α leads to the downregulation of claudin-5 and ZO-1, via PKCζ activation [91]. Cytokine IL-1B, which is found in high levels in the diabetic rat retina, induces iBRB breakdown by leukocyte recruitment and histamine release [92], whereas chemokine CCL2, which was found to be higher in the vitreous of DR patients [93], recruits monocytes, which in turn secrete growth factors and pro-inflammatory mediators in a positive feedback manner [94]. Under hyperglycaemic conditions, leukocyte adhesion molecules such as ICAM1 are upregulated, and metalloproteinases, such as MMP-9 and MMP-2, are activated and cause the shedding of the glycocalyx, which covers the luminal surface of ECs and prevents the adhesion of leukocytes and platelets [95,96].

Pericyte loss, considered an early hallmark of DR, contributes to alterations in iBRB structural integrity and precedes other vascular abnormalities, such as the formation of acellular capillaries [97]. Pericyte dropout from vascular capillaries leads to EC dysfunction, with consequent vascular leakage, blood vessel dilation, and formation of microaneurysms [58,98,99]. The molecular mechanisms underlying pericyte loss remain elusive. However, several mechanisms—including the formation and accumulation of AGEs [100], inflammation, ROS production [101,102], and related signalling pathways—have been correlated with pericyte loss in DR. Pericyte death in diabetes may also result from an uncontrolled immune response, as illustrated by an in vitro study demonstrating that retinal autoantibodies can induce pericyte death by activating the complement system [103].

Finally, structural alterations of the basement membrane are observed in the retinal vasculature of both diabetic animals and patients [65], impairing communication between ECs and pericytes and EC autoregulation.

When enhanced permeability persists, fluid accumulation in the retina overpowers reabsorption mechanisms, leading to the development of DMO.

## 5. Calcium Signalling in the Blood–Retinal Barrier

A growing number of investigations unravelled the role of endothelial Ca^2+^ signalling at the blood–brain barrier (BBB) [104,105,106,107], which plays a similar role as the iBRB by maintaining the proper microenvironment for neurons to function. At the BBB, an array of intra- and intercellular Ca^2+^ signals enable microvascular ECs to detect neuronal activity and synaptic transmission, to regulate local changes in cerebral blood flow, to influence BBB permeability, and to undergo angiogenesis [104,107,108,109,110]. Endothelial Ca^2+^ signals can be triggered by inositol-1,4,5-trisphosphate (InsP_3_)-dependent Ca^2+^ release from the endoplasmic reticulum (ER) [111,112] and lysosomal Ca^2+^ mobilisation through Two-Pore Channels (TPCs) or Transient Receptor Potential (TRP) Mucolipin 1 (TRPML1) channels [113,114], whereas the following reduction in the ER Ca^2+^ concentration leads to the activation of store-operated channels (SOCs) [114]. SOCE in cerebrovascular ECs has been associated with STIM and Orai proteins [115], which underpin the majority of endothelial SOCs throughout peripheral circulation [116]. TRP channels provide an additional means for Ca^2+^ entry pathway at the BBB [104,105]. TRP channels are non-selective cation channels which are, with a few exceptions, permeable to both Na^+^ and Ca^2+^, thereby regulating the endothelial function by inducing both membrane depolarisation and an increase in intracellular Ca^2+^ concentration ([Ca^2+^]_i_) [104,105,117,118]. Endothelial TRP channels at the BBB can be gated by G_q_ protein-coupled receptors (G_q_PCRs) [119,120], neuronal activity [110], reactive oxygen species [121], and dietary agonists [115]. Finally, cerebrovascular ECs are sensitive to laminar shear stress, which causes an increase in [Ca^2+^]_i_ by activating the mechanosensitive Piezo 1 channels [122]. Conversely, the role of endothelial Ca^2+^ signalling at the iBRB is yet to be fully understood [38] and will be the subject of this section.

### Agonist-Induced Ca^2+^ Signals at the iBRB

The resting Ca^2+^ concentration in vascular ECs ranges between 50 and 100 nM [123,124,125] and is maintained by an intricate network of Ca^2+^ transporting systems that include the following: plasma membrane Ca^2+^-ATPase (PMCA), Na^+^-Ca^2+^ exchanger (NCX), Sarco-Endoplasmic Reticulum Ca^2+^-ATPase (SERCA), and mitochondrial Ca^2+^ uniporter (MCU) [20,126]. The Ca^2+^ clearing machinery also intervenes to fine-tune the spatiotemporal organisation of the intracellular Ca^2+^ signals [127,128] and to restore the [Ca^2+^]_i_ to the basal levels upon the removal of an extracellular signal [129,130]. Only SERCA2 was detected at the iBRB [131], while SERCA1 and SERCA3 are unlikely to be expressed [132]. Similarly, it is unknown which PMCA and NCX isoforms are expressed and how they contribute to endothelial Ca^2+^ dynamics at the iBRB. The molecular mechanisms shaping the Ca^2+^ response to physiological agonists are also still unclear. Endothelial agonists stimulate either G_q_-protein-coupled receptors (G_q_PCRs; e.g., acetylcholine, ATP, histamine, glutamate) or tyrosine kinase receptors (TKRs; vascular endothelial growth factor receptor 2 or VEGFR2) to recruit, respectively, phospholipase Cβ (PLCβ) and PLCγ. PLC signalling, in turn, leads to the production of InsP_3_ and diacylglycerol (DAG) from the membrane-bound precursor phosphatidylinositol 4,5-bisphosphate (PIP_2_) [20,133]. InsP_3_Rs are expressed [134], and G_q_PCR activation with the endothelial autacoid, ATP, can stimulate InsP_3_ production [135] in retinal ECs. A recent investigation revealed that type 1 InsP_3_R (InsP_3_R1) supports the ER-to-mitochondria Ca^2+^ shuttle, which plays a crucial role in the regulation of endothelial bioenergetics [127], in human retinal ECs [136]. Interestingly, InsP_3_R1 is physically tethered to the voltage-dependent anion channel 1 (VDAC1) by the glucose-regulated protein 75 (GRP-75) and leads to an MCU-dependent increase in mitochondrial Ca^2+^ concentration [136]. It is, however, still unclear whether InsP_3_R2 and InsP_3_R3, which support ER Ca^2+^ release at the BBB [107], are also expressed at the iBRB. Similarly, the role of TPCs and TRPML1 at this location remains unclear. The purinergic receptor P2 × 4, which was also sporadically detected in acidic vesicles [137], can support retinal neo-angiogenesis [138] but whether this is due to lysosomal Ca^2+^ mobilisation is yet to be demonstrated.

The retinal angiogenesis assay has long been used as a proxy of the physiological roles played by specific components of the Ca^2+^ machinery previously identified in cultured vascular ECs. VEGF has long been known to stimulate sprouting angiogenesis through an increase in endothelial [Ca^2+^]_i_ [133]. In accord, VEGF induces intracellular Ca^2+^ oscillations in human cultured retinal ECs by activating VEGFR2 [139,140,141]. This Ca^2+^ signature strongly resembles the Ca^2+^ signal induced by VEGF in circulating endothelial colony forming cells (ECFCs), which is shaped by ER Ca^2+^ release through InsP_3_Rs and lysosomal Ca^2+^ mobilisation through TPCs and maintained by SOCE [142,143]. It was suggested that the rhythmic ER Ca^2+^ release through InsP_3_Rs is triggered by lysosomal Ca^2+^ release through the Ca^2+^-induced Ca^2+^ release mechanism, according to the “trigger hypothesis” formulated by Galione and Churchill [144]. Lysosomal TPCs are gated by the intracellular second messenger, nicotinic acid adenine dinucleotide phosphate (NAADP), as also shown in cerebral microvascular endothelial cells [108].

In our opinion, further work is mandatory to assess whether the blockade of InsP_3_Rs [118], as well as of TPCs [145], affects VEGF-induced Ca^2+^ waves at the iBRB. The only indirect evidence that VEGF stimulates InsP_3_-induced ER Ca^2+^ release in retinal capillary ECs was provided by Galeano-Otero and coworkers, who showed that the pharmacological blockade of SOCE with GSK-7975A impaired retinal angiogenesis in mouse xenografts [146]. GSK-7975A is a reliable blocker of Orai1, which contributes the Ca^2+^-permeable pore subunit to most endothelial SOCs [116]. According to this model [147,148,149], ER Ca^2+^ release through InsP_3_Rs activates STIM proteins, which detect the fall in free ER Ca^2+^ and translocate to ER–plasma membrane junctions to bind to and gate Orai1. Therefore, the retinal angiogenic assay suggests that both InsP_3_Rs and SOCE shape VEGF-induced Ca^2+^ signals in retinal capillary ECs. Future work on cultured retinal capillary endothelial cells is required to confirm this hypothesis, which could hold promising therapeutic perspectives for DR (see next paragraph).

## 6. Calcium Signalling in Diabetic Retinopathy

The dysregulation of endothelial Ca^2+^ signalling has long been associated with both cardiovascular disorders [22,129,150,151,152,153], e.g., hypertension, type 2 diabetes, atherosclerosis, obesity, sepsis, and deep vein thrombosis, and cancer [154,155]. The endothelial Ca^2+^ machinery at the BBB can also be impaired in brain disorders, including Alzheimer’s disease [156,157,158], ischaemic stroke [159], and traumatic brain injury [160]. It is, therefore, not surprising that the endothelial Ca^2+^ toolkit is remodelled in DR.

### 6.1. ER-Dependent Ca^2+^ Signalling at the iBRB Is Impaired by DR

A recent investigation showed that InsP_3_R1–GRP–75–VDAC1 signalling was enhanced in in vitro models of DR that were obtained by culturing human retinal capillary ECs in the presence of hyperglycaemia or advanced glycosylation end products. This in turn led to mitochondrial Ca^2+^ overload followed by exaggerated ROS production, decreased mitochondrial membrane potential, cytochrome c release, caspase-3 activation, and apoptosis [136]. The ER-to-mitochondria Ca^2+^ shuttle is driven by the basal activity of PLCβ that occurs even in the absence of extracellular stimulation [161]. Intriguingly, the Food and Drug Administration (FDA)-approved drug, LiCl, which inhibits PLC [162], was recently proposed as an adjuvant for anti-VEGF therapy in patients affected by DR [163]. Therefore, the InsP_3_R1–GRP-75–VDAC1 signalling pathway could also be probed in in vivo models of DR to assess its suitability as an alternative target to treat DR patients showing resistance to anti-VEGF therapies. A pioneering investigation suggested that PLC-dependent InsP_3_ synthesis was not affected under high glucose conditions [135]. However, a subsequent study reported that hyperglycaemia can enhance the expression of Gα_q_/PLCβ-mediated signalling through the mitogen-activated protein kinase (MAPK)/phosphatidylinositol 3-kinase (PI3K) pathway [164]. Therefore, future work is mandatory to confirm whether the formation of mitochondria-associated ER membranes (MAMs), which provide the physical substrate for the ER-to-mitochondria Ca^2+^ shuttle, is enhanced by hyperglycaemia. It should also be noted that the exaggerated oxidative burst of single mitochondria can mediate the retrograde activation of the ROS-sensitive InsP_3_Rs [165,166], thereby boosting ER Ca^2+^ release. Therefore, we suggest that agonist-induced Ca^2+^ mobilisation through InsP_3_Rs could be increased in DR. This hypothesis could also explain the reported increase in NO production and neurovascular coupling, as InsP_3_ signalling is a primary determinant of endothelial NO synthase (eNOS) at the BBB [111].

In addition to InsP_3_-induced ER Ca^2+^ release, hyperglycaemia could also enhance endothelial SOCE [167], thereby potentially impacting on the angiogenic response to VEGF. Early studies showed that VEGF-induced endothelial Ca^2+^ signals are dysregulated in cancer [168] and primary myelofibrosis [169]. An exaggerated Ca^2+^ response to VEGF could contribute to explaining the detrimental effects of VEGF on the iBRB structure in DR. Additionally, unveiling the Ca^2+^ signalling machinery driving VEGF-induced Ca^2+^ oscillations could suggest alternative targets to circumvent the resistance to anti-VEGF therapies. Several FDA-approved drugs were also shown to inhibit SOCE [170,171], and the pharmacological blockade of SOCE was proposed as an anti-angiogenic strategy in cancer patients resistant to VEGF inhibitors [172,173]. In this scenario, assessing the molecular make-up of endothelial SOCs at the iBRB is also mandatory, as Orai1 may not be the sole Ca^2+^-permeable channel gated by STIM proteins in response to the ER Ca^2+^ depletion [116]. In accord, VEGF-induced endothelial SOCE can also be sustained by TRP Canonical 1 (TRPC1) [116] and TRPC4 [174]. Moreover, VEGF-dependent Ca^2+^ entry can be further sustained by DAG-regulated TRP channels, including TRPC3 [118] and TRPC6 [175].

### 6.2. Endothelial TRP Channels at the iBRB

TRP channels are the best-characterised Ca^2+^ entry pathway in retinal capillary ECs [38]. The TRP superfamily includes non-selective cation channels that are variably permeable to monovalent (Na^+^ and K^+^) and divalent (Ca^2+^, Mg^2+^, Zn^2+^, Fe^2+^) cations and are sub-divided into six sub-families based upon their sequence homology: canonical (TRPC1-7), melastatin (TRPM1-8), vanilloid (TRPV1-6), ankyrin (TRPA1), polycystin (TRPP), and TRPML1-3. The TRPP sub-family consists of eight members, but only TRPP2, TRPP3, and TRPP5 can be regarded as true ion channels [176,177,178]. TRP channels modulate the endothelial function by promoting both membrane depolarisation and Ca^2+^ entry, which can in turn induce InsP_3_Rs-mediated ER Ca^2+^ release through Ca^2+^-induced Ca^2+^ release [169,179]. Endothelial TRP channels serve as polymodal sensors that integrate multiple physical and chemical signals generated from both the surrounding microenvironment and the circulating blood flow, including second messengers, e.g., DAG and arachidonic acid (AA), reactive oxygen species, e.g., hydrogen peroxide (H_2_O_2_) and 4-Hydroxynonenal (4-HNE), intracellular ions, e.g., an increase [Ca^2+^]_i_ and a decrease in cytosolic Mg^2+^. Additional endothelial TRP channels are sensitive to dietary agonists, e.g., capsaicin, menthol, and allyl isothiocyanate (AITC); gasotransmitters, e.g., NO and hydrogen sulfide (H_2_S); changes in temperature; and mechanical deformations of the plasma membrane, e.g., laminar shear stress, membrane stretch, and osmotic swelling [166,177,178,180,181,182,183]. TRP channels are also widely expressed at the BBB [105], where they regulate CBF and endothelial permeability. For instance, in mouse microcirculation, capillary endothelial TRPA1 channels are activated by neuronal activity through 4-HNE production, thereby initiating a slow inter-endothelial Ca^2+^ wave that increases CBF by stimulating endothelium-dependent hyperpolarisation (EDH) [184]. Similarly, TRPA1 may be activated by 4-HNE [185] or H_2_S [120] in human cerebrovascular ECs to induce NO release, which is the primary vasorelaxing mechanism in human brain microcirculation [186]. In addition, the endothelial TRPC3 and TRPC4 channels, which are both gated by the second messenger DAG [118], regulate BBB permeability and lead to neuronal disorders when their expression is altered [187,188]. TRPV4 can also regulate the BBB integrity under physiological [189], but not inflammatory [190], conditions. Finally, the mechanosensitive TRPP2 isoform, which serves as a Ca^2+^-conducting pathway in combination with TRPC1, could drive stretch-induced BBB damage upon traumatic brain injury [191]. These pieces of evidence led to the hypothesis of a critical role for TRP channels at the iBRB [38].

### 6.3. The Physiopathological Role of Endothelial TRP Channels at the iBRB

**Figure 3:**
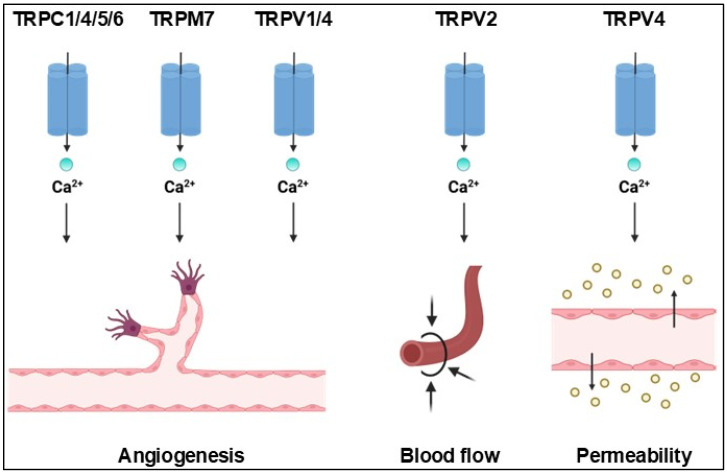
The role of TRP channels in iBRB functions. In retinal ECs, TRPC1/4/5/6, TRPM7 and TRPV1/4 are involved in angiogenesis, whereas Ca^2+^ entry through TRPV4 induces vascular permeability. In VSMCs, TRPV2 regulates blood flow. Created in BioRender. Dragoni, S. (2025) https://BioRender.com/s9eg1dn, accessed on 18 April 2025.

While the role of TRP channels in phototransduction is well understood, their expression and function within the retinal vasculature remain poorly characterised [192]. Despite this limited understanding, existing evidence indicates that TRP channels are critically involved in both normal and disease-related processes in the retinal vasculature (Figure 3). RT-PCR analyses have confirmed the presence of all TRP channel subtypes in the whole mouse retina [193]. Specifically, TRPC1, TRPC3, TRPC4, and TRPC6 have been identified in human retinal ECs, while TRPV1 and TRPV4 are expressed not only in human retinal ECs but also in primary bovine retinal ECs and intact retinal vessels in mice and rats. Additionally, retinal VSMCs in rats express TRPC1, TRPM7, TRPV1, TRPV2, TRPV4, and TRPP1 (Dragoni et al., 2025) (Table 1) [38].

TRPV2—which stimulates angiogenesis and modulates permeability at the BBB [206], where it is the most abundant TRP isoform [207]—is critical in fine-tuning autoregulation at the iBRB (Table 1). As mentioned above, diabetes-related impairments in the mechanism of pressure autoregulation lead to elevated capillary pressure, with consequent EC damage [71]. On the vascular smooth muscle cells of retinal arterioles, TRPV2 channels trigger the myogenic response, thereby contributing to blood flow autoregulation [194]. In diabetic rats, downregulation of TRPV2 and its inability to be activated by stretch impair the myogenic reactivity of retinal arterioles [196]. A decreased expression of TRPV2 was also observed in vascular SMCs from diabetic donors. Interestingly, non-diabetic rats heterozygous for TRPV2, besides lacking the myogenic reaction, also develop the vascular abnormalities typical of DR, such as vascular leakage, formation of acellular capillaries, neovascularisation and upregulation of inflammatory factors [196], suggesting the TRPV2 loss can act as a trigger in the onset of DR.

TRPV4 is another member of the TRPV sub-family that controls both vascular leakage and angiogenesis. TRPV4 can be physiologically activated by laminar shear stress [208] or downstream of the endothelial G_q_PCRs, which stimulate PLCβ to cleave PIP_2_ and gate TRPV4 [119]. Vascular leakage is a critical feature of DR, and it is caused by loss or malfunction of NVU components with consequent iBRB breakdown [209]. It has been established that Ca^2+^ entry plays a pivotal role in the onset of leakage [210]. Several studies have investigated the role of TRPV4 in vascular permeability in health and disease, generating interesting but contradictory results. In diabetic rats, activation of TRPV4 participates in the onset of oedema and TRPV4-selective antagonists resolved iBRB breakdown in diabetic rats (Table 1) [198,199]. From a molecular perspective, TRPV4 activation in human retinal ECs resulted in the degradation of the tight junction protein occludin, the disruption of cortical F-actin and the reduction of VE-Cadherin and β-catenin colocalisation, which were associated with a decrease in the monolayer impedance and consequent increased permeability [211]. These results are consistent with the increase in vascular permeability observed in vivo in WT mice after activation of TRPV4 [198]. However, a different study showed a decrease in TRPV4 expression in both bovine retinal ECs exposed to high glucose and retinal vessels of STZ-induced diabetic rats [200]. It should be noted that contradictory results have always characterised the role of TRPV4 in vascular leakage throughout peripheral and pulmonary circulations. One reason might be related to the different cell and animal models used [211] which showed that TRPV4 can protect [212,213] or disrupt [214,215] the integrity of the endothelial monolayer in different vascular beds or based on the level of channel activation in the different components of the NVU [198,216,217]. TRPV4-mediated Ca^2+^ entry was found to activate eNOS in both mouse [111,119,218] and human [219] cerebrovascular endothelial cells. Future work could assess whether TRPV4 activation at the iBRB also leads to NO production, which could further promote hyperpermeability.

TRPV4 is also critical to retinal angiogenesis in cooperation with TRPV1. Aberrant angiogenesis within the retina leads to the progression of NPDR to PDR [5]. The formation of new blood vessels requires the proliferation and migration of ECs, which then assemble into proper vascular tubes. TRPV1 is less expressed than TRPV4 at the BBB [207], but in the peripheral circulation, TRPV1-mediated Ca^2+^ entry supports the angiogenic sprouting [183]. TRPV1 and TRPV4 are highly expressed in bovine retinal microvascular ECs, with TRPV1 broadly distributed in the cytoplasm and scattered in the plasma membrane (Table 1) [139], and TRPV4 mainly localised in the plasma membrane (Table 1) [211]. Interestingly, a study found that TRPV1 and TRPV4 form heteromeric channels which regulate the tubulogenic phase of the angiogenesis process in vitro and the neovascularisation in vivo in a model of oxygen-induced retinopathy (OIR) [139]. TRPV1 and TRPV4 are sensitive to multiple physiological agonists (Table 1), but the physiological gating signal for the heteromeric TRPV1/TRPV4 channel in bovine retinal ECs is unknown. Interestingly, they are both activated by PIP_2_ depletion (Table 1), thereby potentially placing this Ca^2+^ entry pathway under the control of PLC signalling. A recent investigation demonstrated that TRPV4 is mechanically activated during the pathological neovascularization of the retina [220]. TRPV1 does not serve as a mechanosensory but it could do when assembled with TRPV4.

Retinal angiogenesis can also be supported by TRPC channels, as shown in the remainder of the peripheral circulation [118,221,222]. Indeed, under hyperglycaemic conditions, the expression of TRPC1 and TRPC6 increased, and inhibition of these channels led to a reduction in VEGF expression as well as in proliferation, migration and tube formation of human retinal ECs [202]. Consistently, *trpc1/4/5/6* quadruple knockdown mice subjected to 30 weeks of hyperglycaemia were protected from the vascular abnormalities of DR, such as pericyte loss and retinal thinning [23]. TRPC1 and TRPC6 were not reported to assemble into a heteromeric channel and, therefore, are likely to serve as independent Ca^2+^ entry pathways (Table 1).

Additionally, the OIR model also suggested the critical role of TRPC4 in VEGF-induced angiogenesis [203]. Injection of siRNA against mTRPC4 at P12 in OIR mice decreased retinal neovascularisation and the number of neovascular tufts and avascular areas. At a molecular level, downregulation of TRPC4 via siRNA impaired VEGF-induced migration and tubulogenesis in human retinal ECs and inhibited the activation of the VEGF effectors ERK, p38, and AKT [203]. The mechanism by which VEGF can activate TRPC4 is unclear, but it could involve the STIM1-dependent recruitment of a supermolecular complex including Orai1, TRPC1 and TRPC4, as shown in the pulmonary microcirculation [116,174]. TRPM7, which is sensitive to the intracellular Mg^2+^ concentration [205], has also been involved in retinal angiogenesis, but it is unclear whether it plays any role in retinopathy (Figure 3).

### 6.4. Is There a Link Between Neuroinflammation and Impairment of Ca^2+^ Signalling at the iBRB in DR?

Aberrant Ca^2+^ signalling in astrocytes has long been known as a crucial player of neuroinflammation by initiating inflammatory immune responses following stress, brain injury, or disease-related triggers [223]. Emerging evidence suggested that the hypoxic conditions associated with DR could also increase the spiking in retinal astrocytes [224]. Dysregulated Ca^2+^ signalling could then lead to the massive release of pro-angiogenic factors, pro-inflammatory, and pro-fibrotic mediators that promote neurodegeneration and hypervascularization, thereby favouring DR progression [225]. Additionally, the increased production of ROS that occurs in the retinal microenvironment could promote neurodegeneration by activating ROS-sensitive Ca^2+^ signalling pathways and inducing cytosolic Ca^2+^ overload [226,227]. Intriguingly, excessive Ca^2+^ signalling could in turn further increase redox signalling and exacerbate ROS-induced oxidative stress [227]. Conversely, it is still unclear whether neuroinflammation may interfere with the endothelial Ca^2+^ machinery at the iBRB. Clearly, the massive release of VEGF from gliotic Müller cells will stimulate aberrant angiogenesis through an increase in [Ca^2+^]_i_, as discussed above. Neuroinflammation is also sustained by multiple neurovascular mediators that are released by Müller cells under hyperglycaemic conditions, including interleukin-6 (IL-6) and tumour necrosis factor-α (TNF-α) [225]. IL-6 and TNF-α were shown to increase the resting [Ca^2+^]_i_ in endothelial cells from other vascular beds [228,229,230]. Future work will have to assess whether these pro-inflammatory cytokines can also disrupt the intracellular Ca^2+^ dynamics and the Ca^2+^-dependent functions, i.e., barrier integrity and NO production, at the iBRB. Interestingly, a recent study showed that complement component C3 disrupts the BBB integrity through the C3a/C3aR signalling pathways, which leads to a robust increase in [Ca^2+^]_i_ [231]. The complement pathway plays a critical role in the neuroinflammatory process associated with DR [232], but it is still unknown whether it also affects the intracellular Ca^2+^ homeostasis at the iBRB.

## 7. Conclusions

The clinical causes and molecular pathways driving diabetic retinopathy have been studied for over five decades. Nevertheless, effective and safe therapies are still far away. Intravitreal injections of anti-VEGF and corticosteroids have offered some hope for managing diabetic macular oedema. However, around 50% of patients show little to no benefit from these therapies [13,14,233]. Moreover, laser photocoagulation, which is the current standard approach for treating proliferative diabetic retinopathy, can lead to retinal damage and vision loss. Therefore, new therapeutic options must be investigated.

Dysregulation of Ca^2+^ channels characterises a plethora of diseases, and Ca^2+^ channel blockers are the standard treatment for certain cardiovascular conditions, such as hypertension and arrhythmia. Targeting the intracellular Ca^2+^ toolkit is currently under intense investigation in the search for alternative treatment of life-threatening disorders, such as cancer and severe combined immunodeficiency. Ca^2+^ entry is a pivotal trigger for mechanisms that lead to inner blood–retinal barrier disruption, such as vascular leakage and pathological angiogenesis. Limiting Ca^2+^ channel activity has been shown to be protective against diabetic retinopathy or hyperglycaemia-induced damage. Therefore, further investigation into the role of the components of the Ca^2+^ toolkit may prove to be a promising strategy for identifying new therapeutic targets for diabetic retinopathy and eye diseases. A first mandatory step is the characterisation of the angiogenic Ca^2+^ signalling pathways recruited by VEGF, e.g., InsP_3_Rs, TPCs, SOCE, and/or TRPC channels. The growing availability of FDA-approved drugs that can be repurposed to block Orai1, which is the primary pore-forming subunit of the endothelial SOCs, could then lead to further investigations assessing their efficacy in in vivo models of DR. The following FDA-drugs were shown to inhibit SOCE: flecainide, propranolol, lithium chloride, leflunomide, and teriflunomide [170,171]. Furthermore, CalciMedica developed a selective Orai1 inhibitor, termed Auxora^TM^, that showed a safety profile and tolerability in a Phase 2b clinical trial for the treatment of acute pancreatitis (AP) with accompanying systemic inflammatory response syndrome (SIRS) [234,235]. Additionally, TPCs are also sensitive to several FDA-approved drugs, which were suggested as an alternative approach to prevent SARS-CoV-2 infection during the COVID-19 pandemic [236,237]. The following FDA drugs also target lysosomal TPCs: salmeterol, PF-543, racecadotril [238], pimozide, fluphenazine [239], clomiphene, raloxifene [240], verapamil, diltiazem, and nimodipine [240]. Then, it will be worth of assessing whether endothelial TRP channels could also serve as an effective molecular target to treat DR. Many endogenous mediators and synthetic compounds were found to serve as agonists or inhibitors of TRP-mediated Ca^2+^ signals. TRPV2 loss could be, at least partially, rescued by TRPV2 agonists, such as cannabidiol and the anti-gout medication probenecid [206,241]. Excessive Ca^2+^ entry through TRPV4 channels could be hampered by the selective antagonist GSK2798745 [242]. Phase 2 clinical trials demonstrated that GSK2798745 showed a benign safety profile in healthy volunteers and in patients suffering from cardiogenic lung oedema (https://clinicaltrials.gov/study/NCT02497937?term=trpv4&rank=3, accessed on 5 March 2025). Intriguingly, GSK2798745 also blocked TRPV4-mediated Ca^2+^ entry in human retinal microvascular endothelial cells [243]. Therefore, further pre-clinical and clinical investigations could assess whether the manipulation of SOCE or TRP signalling provides an alternative therapeutic strategy for DR. As anticipated, this approach will benefit of the straightforward elucidation of the pro-angiogenic Ca^2+^ signalling machinery recruited by VEGF at the iBRB.

## Figures and Tables

**Table 1:** Endothelial Transient Receptor Potential channels at the inner blood–retinal barrier. AA: arachidonic acid; DAG: diacylglycerol; ERK: extracellular signal-regulated kinase; STIM1: stromal interaction molecule 1; and VSMCs: vascular smooth muscle cells.

TRP Isoform	Cell Type	Agonist(s)	Ca^2+^-Dependent Target	Function in Health and Disease	Ref.
TRPV2	Rat retinal arteriolar VSMCs	Membrane stretch, heat (>52 °C), cannabinoids (e.g., cannabidiol)	Membrane depolarisation and Ca^2+^ entry	Blood flow autoregulation	[194,195,196,197]
TRPP1	Rat retinal arteriolar VSMCs	Membrane stretch	Membrande depolarisation and Ca^2+^ entry (to be confirmed)	Blood flow autoregulation (to be confirmed)	[194]
TRPV1	Bovine retinal ECs	PIP_2_ depletion, heat (>43 °C), hydrogen peroxide	Unknown	Angiogenesis	[139,183]
TRPV4	Human, bovine, mouse and rat retinal ECs	PIP_2_ depletion, heat (>27 °C), osmotic stimulation, laminar shear stress, AA and metabolites	Unknown	iBRB permeability ↑, angiogenesis	[119,139,198,199,200,201]
TRPC1	Human retinal ECs	ER Ca^2+^ depletion via STIM1-dependent activation	VEGF production	Retinal angiogenesis	[116,202]
TRPC3	Human retinal ECs	DAG	Unknown	Retinal angiogenesis	[178,202]
TRPC4	Human and mouse ECs	ER Ca^2+^ depletion via STIM1-dependent activation, G_q_α and G_i/o_α	ERK, p38 and AKT signalling pathways	Retinal angiogenesis	[203,204]
TRPC6	Human retinal ECs	DAG	VEGF production	Retinal angiogenesis	[178,202]
TRPM7	Indirect evidence from TRPM7-deficient mice	Reduction in the intracellular Mg^2+^ concentration	Stimulates glycolysis	Retinal angiogenesis	[178,205]

## Data Availability

No new data were created or analyzed in this study.
